# *Paeniclostridium sordellii* and *Clostridioides difficile* encode similar and clinically relevant tetracycline resistance loci in diverse genomic locations

**DOI:** 10.1186/s12866-019-1427-5

**Published:** 2019-03-04

**Authors:** Callum J. Vidor, Dieter Bulach, Milena Awad, Dena Lyras

**Affiliations:** 1 Infection and Immunity Program, Monash Biomedicine Discovery Institute and Department of Microbiology, Monash University, Clayton, Victoria Australia; 2 Melbourne Bioinformatics, The University of Melbourne, Melbourne, Victoria Australia

**Keywords:** Antibiotic resistance, Mobile genetics, *Clostridium*, *Difficile*, *Sordellii*

## Abstract

**Background:**

With the current rise of antibiotic resistance in bacteria, it is important to monitor the efficacy of antimicrobials in clinical use. *Paeniclostridium sordellii* (previously *Clostridium sordellii*) is a bacterial pathogen that causes human uterine infection after spontaneous or medically induced abortion, for which mortality rates approach 100%. Prophylactic antibiotics have been recommended for individuals undergoing medically-induced abortion, one of which is doxycycline, a member of the tetracycline antibiotic family. However, tetracycline resistance had not been well characterized in *P. sordellii*. This study therefore aimed to determine the levels of tetracycline resistance in *P. sordellii* isolates, and to identify associated loci and their genomic locations.

**Results:**

Using a MIC assay, five of 24 *P. sordellii* isolates were found to be resistant to tetracycline, minocycline, and importantly, doxycycline. Analysis of genome sequence data from 46 isolates found that phenotypically resistant isolates encoded a variant of the *Clostridium perfringens* tetracycline resistance determinant Tet P. Bioinformatic analysis and comparison of the regions surrounding these determinants found variation in the genomic location of Tet P among *P. sordellii* isolates. The core genome comparison of the 46 isolates revealed genetic diversity and the absence of dominant genetic types among the isolates. There was no strong association between geographic location of isolation, animal host or Tet P carriage with isolate genetic type. Furthermore, the analysis of the Tet P genotype revealed that Tet P is encoded chromosomally, or on one of two, novel, small plasmids, all consistent with multiple acquisition and recombination events. BLAST analysis of *Clostridioides difficile* draft genome sequences also identified a Tet P locus, the genomic location of which demonstrated an evolutionary relationship with the *P. sordellii* locus.

**Conclusions:**

The Tet P determinant is found in variable genomic locations within diverse human and animal isolates of *P. sordellii* and *C. difficile*, which suggests that it can undergo horizontal transfer, and may disseminate tetracycline resistance between clostridial species. Doxycycline is a suggested prophylactic treatment for *P. sordellii* infections, however, a small sub-set of the isolates tested are resistant to this antibiotic. Doxycycline may therefore not be an appropriate prophylactic treatment for *P. sordellii* infections.

**Electronic supplementary material:**

The online version of this article (10.1186/s12866-019-1427-5) contains supplementary material, which is available to authorized users.

## Background

With the increase in antibiotic resistance among bacterial populations there is a growing need to develop new antimicrobial drugs, and to investigate the efficacy of those already in use. A family of antimicrobials that have had broad-spectrum applications are the tetracyclines [1]. Tetracyclines are a family of compounds that bind to the 30S ribosomal subunit of the bacterial ribosome and block protein synthesis through inhibition of tRNA binding [1]. However, due to the widespread use of tetracyclines, many resistance determinants are now found in diverse bacterial species [2]. These tetracycline resistance determinants are often associated with mobile genetic elements and act via two main mechanisms [2, 3]. The first mechanism utilizes efflux proteins of the major facilitator superfamily that actively pump tetracyclines out of the bacterial cell [4]. The second mechanism involves ribosomal protection proteins (RPPs) [5], which bind to the bacterial ribosome, leading to a conformational change and subsequent dissociation of the tetracyclines [4]. While only one type of determinant is generally required to provide tetracycline resistance, phenotypic differences can be observed depending on the type of tetracycline administered. For example, RPPs can also protect against minocycline whereas an efflux protein may not [6].

Despite the widespread emergence of tetracycline resistance, this antibiotic family is still being used for the treatment of specific conditions such as infections with *Brucella* or *Chlamydia* species [7, 8]. They are also recommended for the prophylaxis of infection against organisms known to cause intrauterine infections, including those caused by *Paeniclostridium sordellii* [9]. *P. sordellii*, previously known as *Clostridium sordellii*, is a Gram-positive pathogen that causes severe oedemic, myonecrotic or enterotoxic infections in humans, and farm animals including foals, sheep and cattle [10]. Of particular concern over the last 20 years is an association of *P. sordellii*-mediated uterine infection post childbirth, spontaneous abortion and medically induced abortion, with the drug misoprostol [10, 11]. While rare, in each of these cases patients have displayed a sudden infection onset, leading to mortality rates approaching 100% [9, 10]. These cases, along with other causes of post-abortive infection, have led to expert bodies recommending antibiotic prophylaxis prior to surgical and medical abortion [9]. Some of these recommendations include the use of the tetracycline family antibiotic doxycycline, despite levels of tetracycline resistance not being well characterized in *P. sordellii* [9].

In general, *P. sordellii* strains are assumed to be tetracycline sensitive [10, 12, 13], despite previous studies showing that tetracycline resistance occurs in this organism [14, 15]. One study in particular identified three *P. sordellii* isolates from cattle with malignant oedema that were resistant to oxytetracycline, with subsequent PCR analysis indicating that all three isolates carried the *tetA*(P) and *tetB*(P) tetracycline resistance genes [14]. These genes are found in *Clostridium perfringens* within the Tet P determinant (Additional file 1: Figure S1) [6], although their presence in isolates of *Clostridium septicum* and *Clostridioidies difficile* has also been reported [14, 16]. The Tet P determinant is comprised of two genes: *tetA*(P), which encodes a tetracycline efflux protein, and *tetB*(P), which encodes a ribosomal protection protein [6] (Additional file 1: Figure S1). The genes are transcriptionally linked and have an overlap of 17 bp in their protein coding regions. In *C. perfringens*, the Tet P determinant has only been characterized on large conjugative plasmids related to the plasmid pCW3 [17, 18]. Two genes are found downstream of Tet P on *C. perfringens* pCW3- like plasmids, specifically, *pcw303* that encodes a product of unknown function, and *regA*, which encodes a putative AraC-like transcriptional regulator [19, 20]. While it appears that Tet P is present in other clostridial members, the genomic context of this locus and any association with mobile genetic elements is unknown.

Here we determined the tetracycline resistance profiles of a diverse group of *P. sordellii* isolates and identified the genes and genomic location of their tetracycline resistance loci. Using MIC assays, we identified five of 24 isolates that were resistant to three common members of the tetracyclines. BLAST analysis of the genome sequences of these five tetracycline resistant (Tet^R^) isolates identified the Tet P determinant in all of them. Bioinformatic analysis of these genome sequences, along with all publically available *P. sordellii* genomes, showed that Tet P is not carried on pCW3-like plasmids as seen in *C. perfringens* but instead is found in highly variable locations on either the chromosome or on small plasmids. Analysis of *C. difficile* genome sequences also identified Tet P loci and has revealed a link with the Tet P loci observed in *P. sordellii*.

## Results

### Diverse *P. sordellii* strains are resistant to tetracyclines

**Fig. 1:**
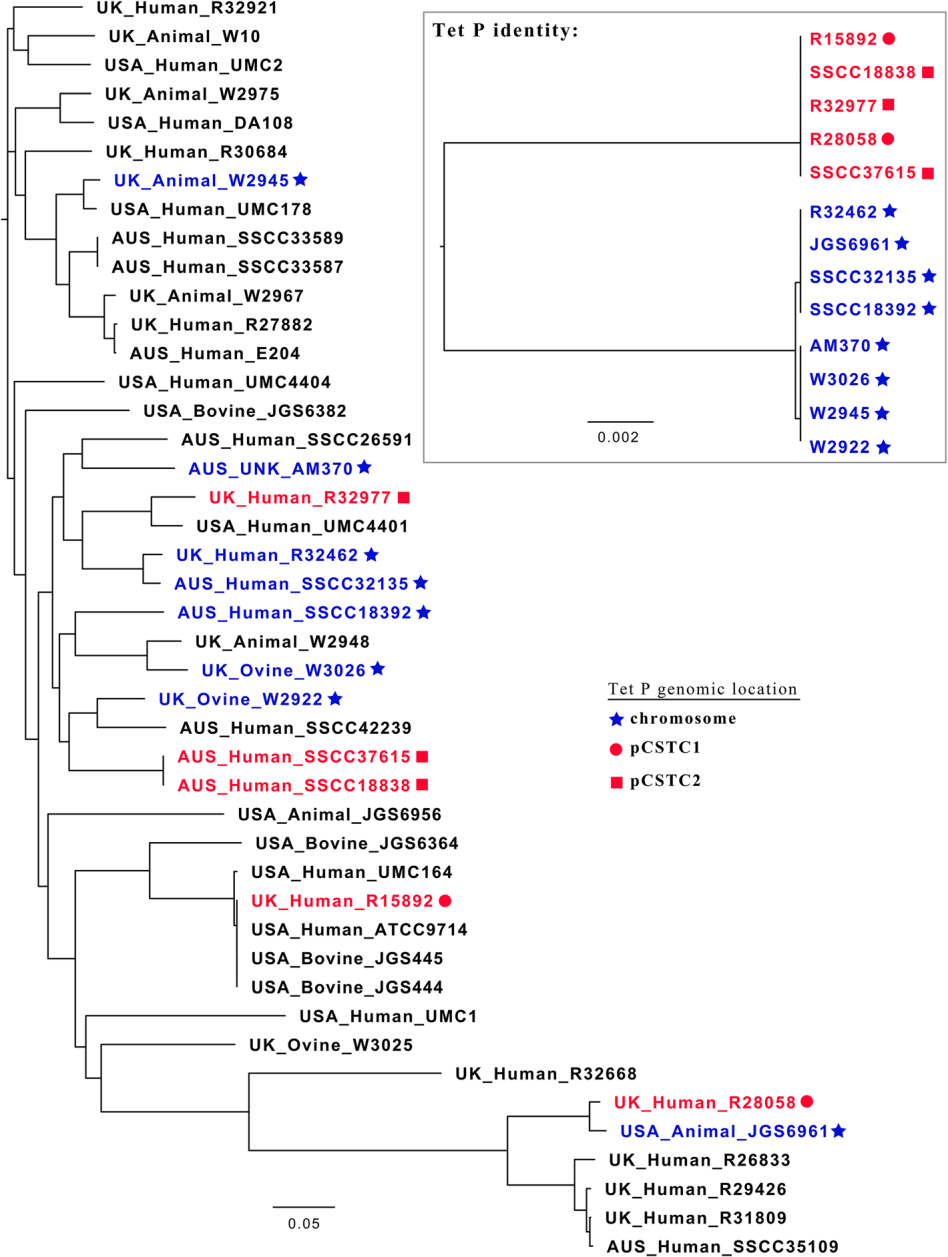
Core SNP phylogeny of *P. sordellii* isolates displaying Tet P genomic location and subtype. A core SNP phylogeny tree of 44 *P. sordellii* isolates was produced from whole genome sequencing reads (see Table S2 for accession numbers) by comparison with the genome sequence of *P. sordellii* type strain ATCC 9714 (LN679998, LN679999 and LN680000) using the Nullarbor pipeline. Shown in the top right corner is a tree representing the nucleotide identity between the Tet P determinants of encoding isolates. The Tet P sequences clearly fall into two main subtypes, and *P. sordellii* isolates that carry Tet P are coloured either in red (sub-type 1) or in blue (sub-type 2). The predicted genomic location of Tet P based on bioinformatic analysis is indicated with a shape next to each encoding strain; chromosomal (star), pCSTC1 (circle) or pCSTC2 (square) (Fig. 2 a, b)

To test if tetracycline resistance is present in *P. sordellii*, a resistance profile was determined for 24 geographically diverse human and animal isolates (Additional file 2: Table S2). Resistance to tetracycline, minocycline and doxycycline was examined in these isolates in comparison to positive and negative *C. perfringens* controls (Table 1). Of the 24 *P. sordellii* isolates, five displayed much higher minimum inhibitory concentration (MIC) values for all three antibiotics when compared to the other *P. sordellii* isolates and the *C. perfringens* negative control (Table 1). The level of resistance to the tetracyclines appeared similar for most of the *P. sordellii* isolates, however, isolate R28058 displayed MIC values two to four times greater than those of the other four resistant isolates (Table 1). R28058 is the only phenotypically Tet^R^ isolate that belongs to the most distant clade of *P. sordellii* (Fig. 1) [21], thus the increased resistance to the tetracyclines in this isolate may reflect intrinsic isolate differences that impact on the resistance phenotype.

**Table 1:** Tetracycline resistance profiles of clostridial isolates

Isolate	MIC (mg/L)		
	Tetracycline	Minocycline	Doxycycline
*P. sordellii*			
Tet^S^ isolates (*n* = 18)	< 0.125	< 0.047	< 0.047
SSCC37615	8	1.5	1.5–3
SSCC18838	8–16	1.5–3	1.5–3
SSCC18392	8–16	3	3
SSCC32135	8	1.5	1.5–3
UMC2	< 0.125	< 0.047	0.094
R28058	32	6	6
*C. perfringens*			
JIR4 (+ control)	8–16	1.5–3	3
JIR325 (− control)	< 0.125	< 0.047	0.094
*C. difficile*			
630 (+ control)	> 32	12	N/A
CD37 (− control)	< 0.125	< 0.094	N/A
MCD43	4	0.375	N/A
MCD46	2–4	0.375	N/A

Displayed is the MIC value (in mg/L) determined for tetracycline, minocycline and doxycycline using a microdilution method across two independent experiments for *C. difficile* and three for the other species. If different values were determined for MICs between experiments they are represented as a range between those values. Clostridial isolates are listed with their species and isolate name. Tet^S^ isolates refers to the remaining 18 isolates of *P. sordellii* tested that all displayed sensitivity to the lowest concentration of each antibiotic tested (see Additional file 2: Table S2)

While obtained from a small sample size, the finding that ~ 20% of the *P. sordellii* isolates tested are resistant to tetracyclines is of concern because doxycycline has been recommended for prophylaxis against post-abortive uterine infections, including those caused by *P. sordellii* [9]. A recent screening of women for vaginal and rectal carriage of *P. sordellii* and *C. perfringens* concluded that both organisms were transient and occurred at a low rate and therefore screening or prophylactic approaches were not advisable [22]. However, due to the severe nature of infection with these organisms, and the fact that prophylaxis does not solely target the clostridia [9], it may be that alternatives to doxycycline should be used for prophylaxis prior to abortion, such as metronidazole or metronidazole combined with doxycycline, as has been recommended by other expert agencies [9]. The collection of a larger number of isolates followed by antibiotic susceptibility testing is needed to fully characterise the burden of tetracycline resistance among the *P. sordellii* population.

### Tetracycline resistance in *P. sordellii* is encoded by a variant of the *C. perfringens* Tet P determinant

The genome sequencing data for 46 *P. sordellii* isolates, which included the 24 isolates used in our antibiotic resistance screening, were analysed for the presence of tetracycline resistance genes. Preliminary evaluation of the quality of the publically available data sets revealed two isolates, W2946 and W2947, were not mono-isolates and were removed from the analysis. All five Tet^R^ isolates as determined by MIC encoded a variant of the *C. perfringens* tetracycline resistance determinant, Tet P (Additional file 1: Figure S1). Analysis of the *P. sordellii* genomic data detected a further eight isolates that carried a Tet P locus, which is therefore present in ~ 30% of isolates tested. This determinant was absent in the genome sequences of all 19 phenotypically tetracycline sensitive (Tet^S^) *P. sordellii* isolates, with the exception of R15892. PCR analysis of two independent stocks of R15892 could not detect Tet P. We therefore believe that although the published genome sequence of R15892 contains Tet P, the isolate used in the MIC assay has lost this element. This is further validated by our analysis of the genome sequence of this isolate that indicates Tet P is encoded on a plasmid; therefore if the isolate lost the plasmid it may lose its resistance to tetracycline.

In the 13 Tet P-containing *P. sordellii* isolates, *tetA*(P) and *tetB*(P) were the same size and arranged as seen in *C. perfringens* (Additional file 1: Figure S1). The coding sequences of the genes had ~ 88.3–89.5% and ~ 96.5–97.9% identity to TetA(P) and TetB(P) from *C. perfringens*, respectively (Additional file 3: Table S1). Sequence comparison of the 13 Tet P loci found in the *P. sordellii* isolates identifies two distinct locus types (Fig. 1, Additional file 3: Table S1), with five isolates carrying subtype 1 (Fig. 1, red) and the remaining eight isolates carrying subtype 2 (Fig. 1, blue). The upstream regulatory sequences present for Tet P in *C. perfringens* are observed in the majority of *P. sordellii* isolates (Additional file 3: Table S1), including the P3 promoter and T1 transcriptional repressor sequence (Additional file 4: Figure S2) [23]. While the sequence of the upstream regions differs between the two *P. sordellii* Tet P locus subtypes, sequence identity is observed within the subtypes (Additional file 3: Table S1), although isolate W2945 displays a deletion which has resulted in the absence of T1 (Additional file 4: Figure S2). The lack of T1 may cause an upregulation of Tet P transcription [23], however, further studies are required to examine this hypothesis.

### The genomic location of Tet P in *P. sordellii* isolates is highly variable

**Fig. 2:**
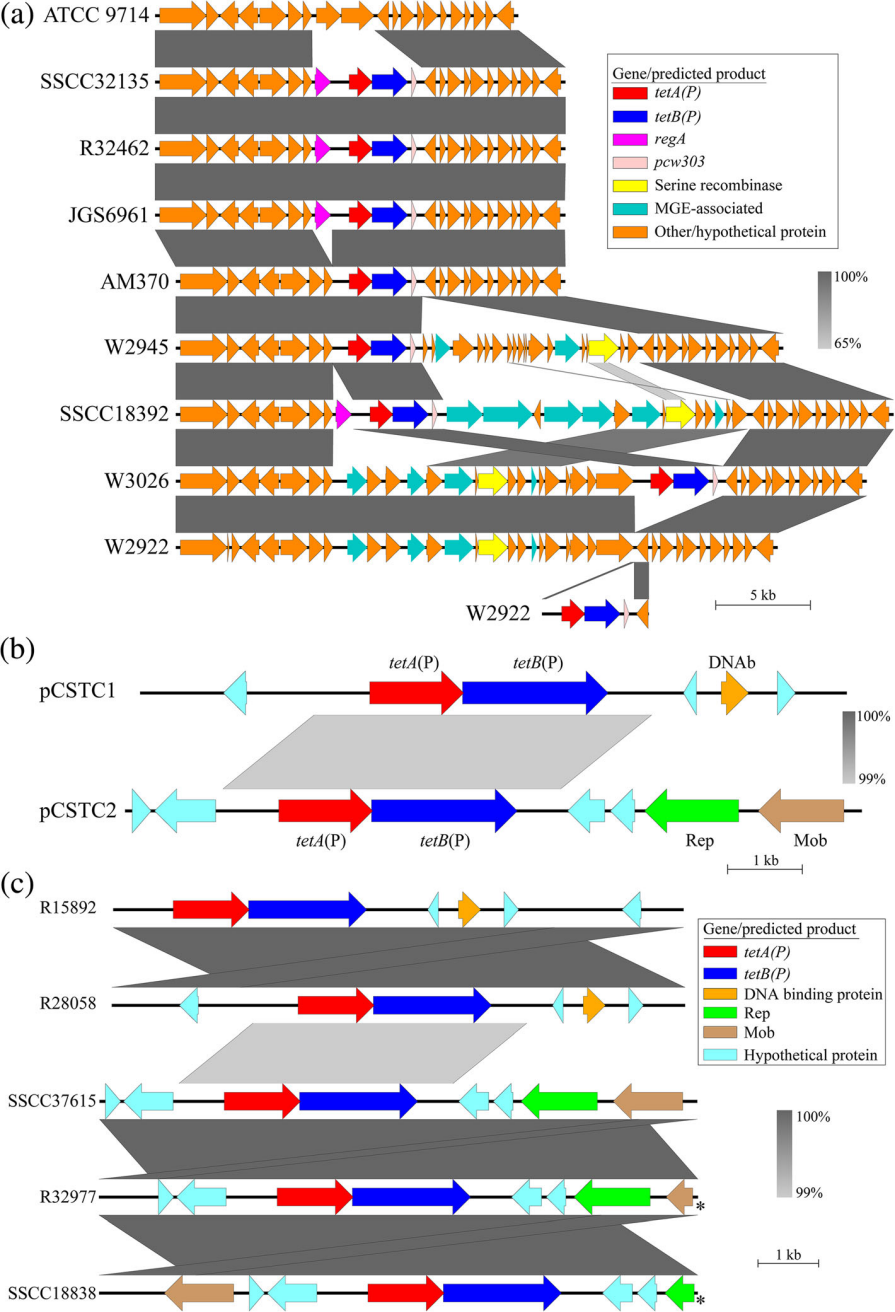
The variable genomic locations of Tet P among isolates of *P. sordellii*. Shown are blastn alignments of *P. sordellii* isolates that encode Tet P or isolates with related loci. Regions of nucleotide identity between sequences are represented by grey bars; the higher the identity, the darker the grey, as illustrated by the legends. The cut off for nucleotide identity was a maximum e value of 0.001 with no minimum identity values over a length of 150 bases. Produced using EasyFig [43]. **a** *P. sordellii* isolates that encode Tet P on the chromosome in comparison to the related genomic location in isolates ATCC 9714 and W2922. ORFs are coloured according to known/predicted function (refer to key). **b** The novel *P. sordellii* tetracycline resistance plasmids pCSTC1 and pCSTC2. The sequence for pCSTC1 is from isolate R28058 but is also present in R15892 (see Fig. 2c). The sequence for pCSTC2 is from isolate SSCC37615 but is also found in isolates R32977 and SSCC18838 (see Fig. 2c). ORFs shown in light blue encode hypothetical proteins, ORFs shown in colour are annotated with their gene name or the predicted function of their product; tetracycline resistance genes *tetA*(P) (red) and *tetB*(P) (blue), putative DNA binding protein (DNAb) (orange), plasmid replication protein (Rep) (green) and conjugative relaxase (Mob) (brown). **c** Comparison of the Tet P containing plasmid sequences identified in *P. sordellii* isolates. ORFs are colored according to the gene they represent or the predicted function of their product (refer to key). ORFs truncated due to a break in the contig are marked with ‘*’

In eight of the 13 Tet P^+^ *P. sordellii* isolates, the determinant was found on large contigs predicted to be part of the chromosome (via a comparison with the partially closed genome sequence of the *P. sordellii* type strain ATCC9714 (Fig. 2a) and the presence of chromosomal genes in flanking regions). These isolates carry Tet P in place of two putative genes encoding proteins of unknown function when compared to the genome sequence of ATCC9714 (Fig. 2a), however, the regions directly flanking Tet P in these eight isolates showed significant variation (Fig. 2a). Three isolates (SSCC32135, JGS6961 and R32462) only contain Tet P flanked by the pCW3 homologs *regA* (purple) and *pcw303* (pink) (Additional file 1: Figure S1, Fig. 2a), however, the presence of *regA* upstream of Tet P in *P. sordellii* differs to that of *C. perfringens*. The product of *regA* is a predicted AraC-like inducible regulator, hypothesized in *C. perfringens* to play a role in the inducible tetracycline phenotype observed in some isolates [19], while no function can be predicted for the product of *pcw303*. While the conservation of *regA* between Tet P carrying isolates of *P. sordellii* and *C. perfringens* suggests that it may play some role in tetracycline resistance, nine of the 13 Tet P^+^ *P. sordellii* isolates do not encode RegA anywhere on the genome, suggesting that RegA is not required for tetracycline resistance. The absence of *regA* in four of the isolates that chromosomally encode Tet P (Fig. 2a) may be explained by the presence of a 19 bp direct repeat that also flanks *regA* in all remaining isolates and which could serve as a homologous recombination site that leads to the loss of this gene (Additional file 5: Figure S3). Apart from containing pCW3-related genes within it, regions flanking this variable locus among the *P. sordellii* isolates show no relationship to plasmid sequences. These flanking regions are also highly conserved among *P. sordellii* isolates and encode tRNAs, chromosomal DNA partitioning proteins and ribosomal components. We therefore believe that this region of the genome is chromosomal and therefore suggest that horizontally acquired DNA has integrated into the same chromosomal hotspot.

Three isolates that encode Tet P chromosomally (W2945, SSCC32135, and W3026) have numerous ORFs positioned either upstream or downstream of Tet P (Fig. 2a), but these flanking regions show little homology between isolates. There are exceptions however, with a region encoding eight ORFs downstream of Tet P in SSCC18392 that has high identity (~ 96%) to a region upstream of Tet P in W3026 (Fig. 2a). The Tet P associated locus of W3026 is also almost entirely conserved with isolate W2922, however the Tet P determinant is absent, instead being located on a separate contig which is flanked by regions of homology to the site of deletion (Fig. 2a). Analysis of the isolate’s pan-genome did not indicate a mixed isolate. We suspect that the two contigs represent a sub-population of the single sequenced isolate in which the Tet P region has potentially looped out or been deleted and subsequently lost from the cell.

The predicted functions of the products encoded in these variable regions are associated with mobile genetic elements or phage, particularly in isolate SSCC18392 (Fig. 2a). Such putative products include a phage DNA methyl transferase, phage primase, a helicase and a type III restriction modification system protein [24]. Despite the differences between the Tet P flanking regions in each isolate, a common feature is that they each encode a predicted large serine-recombinase (Fig. 2a) [24]. No conserved direct or inverted repeat could be identified within the region that could explain the high variation and apparent genetic rearrangement seen among these isolates, however, the conservation of a large serine recombinase gene among these isolates indicates that its product may play a role in this variation. The potential mobility of this chromosomal Tet P locus is further evidenced by their distribution across isolates from distinct clades of *P. sordellii*, and suggests multiple acquisition and rearrangement events among *P. sordellii* isolates (Fig. 1).

The remaining five Tet P^+^ isolates of *P. sordellii* encode Tet P on one of two small plasmids (Fig. 2b, c). The presence of these plasmids and the chromosomal location of TetP in other isolates of *P. sordellii* was confirmed using Southern hybridization on five Tet P^+^ isolates (Additional file 6: Figure S4). The sequence identity between subtypes of Tet P among the population also correlates with genomic location, with sub-type 1 all being plasmid associated and sub-type 2 appearing to be carried on the chromosome (Figs. 1 and 2a, c). Isolates R15892 and R28058 carry Tet P on a novel ~ 9.4 kb plasmid (Fig. 2c), designated pCSTC1 (Fig. 2b). Putative functions could not be assigned to the other four small ORFs on pCSTC1, however pCSTC1_002 contains a predicted C-terminal DNA/RNA-binding domain [25] that may encode a replication initiation (Rep) protein. The remaining three isolates (SSCC37615, SSCC18838 and R32977) encode Tet P on a novel ~ 9.8 kb plasmid (Fig. 2c), designated pCSTC2 (Fig. 2b). Apart from the high region of identity (~ 99%) of Tet P, the two pCSTC plasmids have no significant homology to one another (Fig. 2b). A putative replication gene, *rep*, was identified on pCSTC2 [24], the product of which has 62% identity to the Rep protein from the *C. perfringens* plasmid pIP404 [26]. pIP404 is used as the base replicon for many *C. perfringens* shuttle vectors [27]. An origin of replication with significant identity to that of pIP404 is also present on pCSTC2 [26]. Transformation of *C. perfringens* JIR325 (a Tet^S^ isolate), selecting for Tet^R^, using genomic DNA from SSCC37615, SSCC18838 and R28058 resulted in transformation frequencies of 8.9 × 10^4^, 7.2 × 10^4^ and < 10 cfu/ml (below the detection limit), respectively. This finding indicates that pCSTC2, but not pCSTC1, can replicate in *C. perfringens*, and this was further confirmed using PCR (Additional file 7: Figure S5). These results suggest that an evolutionary relationship exists between pIP404-like plasmids and pCSTC2.

A predicted MobA/MobL family domain (pfam03389) protein is encoded on pCSTC2 (Fig. 2b) [24]. This family of proteins includes MobA from the *E. coli* plasmid RSF1010, a relaxase that initiates transfer of this plasmid to a recipient cell [28]. The presence of a Mob protein indicates that pCSTC2 may undergo conjugative transfer, thereby transferring tetracycline resistance to sensitive *P. sordellii* isolates. Bacterial matings were performed using SSCC37651 (pCSTC2), SSCC18838 (pCSTC2) and R28058 (pCSTC1) as donors with Tet^S^ *P. sordellii* and *C. perfringens* isolates. No transconjugants could be detected under the conditions tested, and therefore the mobility of either pCSTC plasmid could not be confirmed during this study. However, by examining the relatedness of strains that carry these plasmids there is evidence of their mobility. Isolates that carry pCSTC1 or pCSTC2 are not all clonal, but are instead found in distantly related isolates (Fig. 1). The fact that isolates from distinct clades encode identical plasmids suggests a recent acquisition event, thereby indicating that both Tet P-encoding plasmids may be able to undergo mobilisation between *P. sordellii* strains.

### Tet P is found in human clinical isolates of *C. difficile*

Recent work has detected Tet P in three ribotype 0014 porcine *C. difficile* isolates [16]. The genomic location of Tet P in all isolates was found to be identical and not associated with plasmid or mobile genetic element-associated genes [16]. We looked for Tet P among draft genome sequences of *C. difficile* isolates obtained from an Australian hospital. Two isolates, MCD43 and MCD46, contained the Tet P determinant within contigs that were ~ 49.9 kb and ~ 123.5 kb in length, respectively. The nucleotide sequence identity between these contigs was 100%. The predicted coding sequences of TetA(P) and TetB(P) from MCD43/46 were identical to those of the porcine *C. difficile* isolates, as well as being identical to *P. sordellii* Tet P subtype 2 (Additional file 3: Table S1). The upstream Tet P regulatory region of MCD43/46 was identical to that of *P. sordellii* W2945, including the deletion of the T1 transcriptional terminator (Additional file 4: Figure S2). This region in *C. difficile* porcine isolates, despite the presence of a few SNPs, was highly related to that of *P. sordellii* Tet P subtype 2-encoding strains and unlike MCD43/46 contained the T1 transcriptional terminator (Additional file 3: Table S1, Additional file 4: Figure S2).

**Fig. 3:**
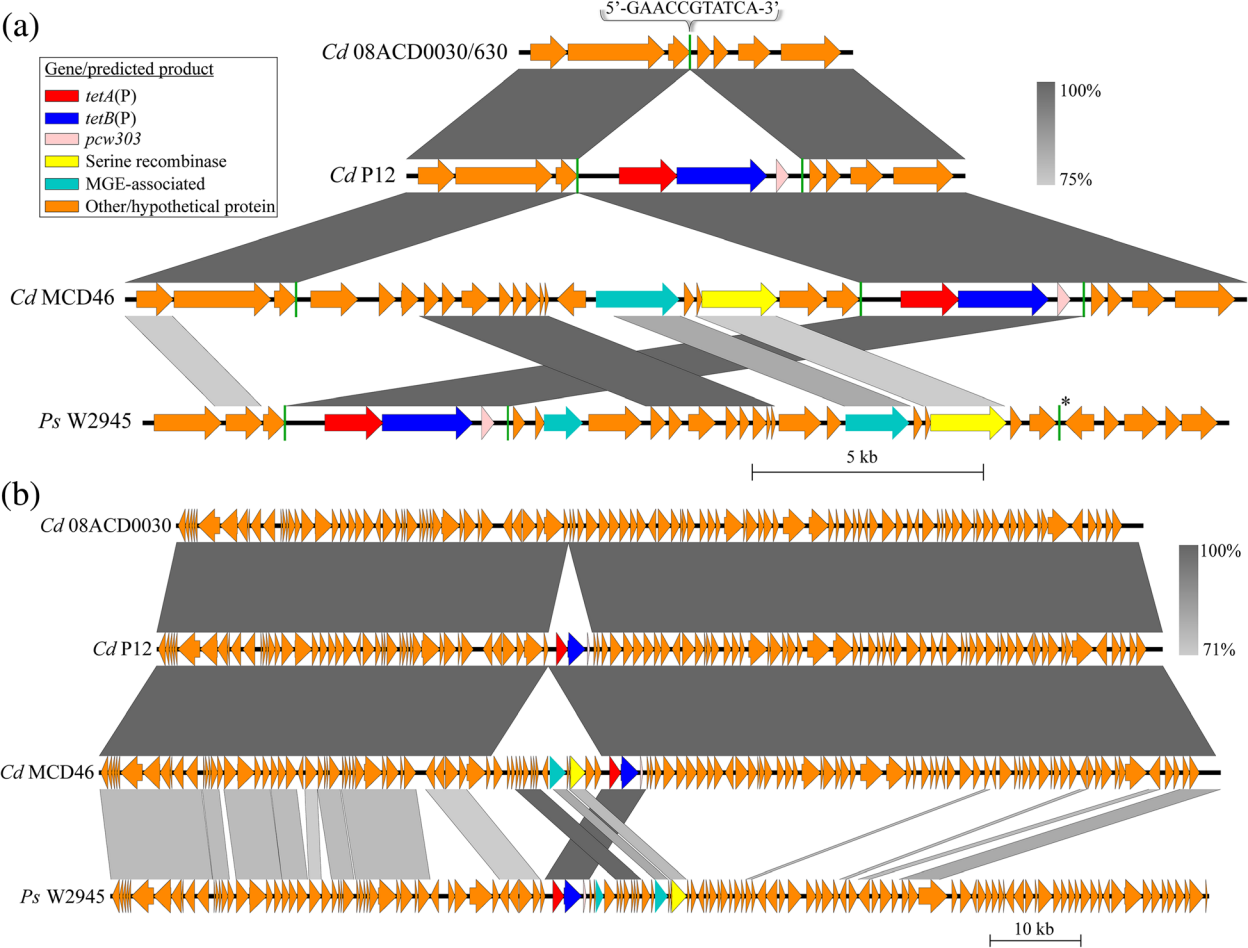
The chromosomal location of Tet P in isolates of *C. difficile*. Shown are visual representations of blastn alignments. ORFs are colored according to the gene they represent or the predicted function of their product [refer to key in (a)]. Regions of nucleotide identity between sequences are represented by grey bars; the higher the identity, the darker the grey, as illustrated by the legends. Produced using EasyFig [43]. **a** *C. difficile* (*Cd*) isolates that encode Tet P (P12 and MCD46) in comparison to the equivalent chromosomal location in the Tet P negative isolate 08ACCD0030 (for which this region is 99% identical to that of *C. difficile* type strain 630) and the Tet P^+^ *P. sordellii* (*Ps*) isolate W2945. The cut off for nucleotide identity was a maximum e value of 0.001 with no minimum identity values over a length of 150 bases. The location of an 11 bp region, the sequence for which is shown in the figure, is indicated by green lines on each genome sequence. The one exception is the repeat marked with ‘*’, for which there is a single base pair mismatch. **b** Comparison displaying the greater genomic context for chromosomally encoded Tet P in *C. difficile* isolates. Shown are the *C. difficile* (*Cd*) isolates in comparison to their relative genomic location in the Tet P^−^ isolate 08ACCD0030 and the Tet P^+^ *P. sordellii* (*Ps*) isolate W2945. The cut off for nucleotide identity was a maximum e value of 0.001 with no minimum identity values over a length of 500 bases

The MCD46 contig was compared to Tet P *C. difficile* genomes within GenBank, and a subsequent BLAST alignment was performed between the Tet P locus of this isolate, isolate 08ACD0030 (its most similar hit), type strain 630 and isolate P12, a representative of the porcine isolates that contains Tet P (Fig. 3a, b). The majority of the Tet P containing contigs displayed close to 100% identity to a region within the 08ACD0030 draft genome (Fig. 3b), which was predicted to be chromosomal through a comparison with the closed 630 genome [29]. In the porcine Tet P containing isolates an insertion has occurred that contains Tet P, its upstream regulatory region and *pcw303* (Fig. 3a, Additional file 1: Figure S1). The same insertion was also found in the human Tet P containing isolates, however these genomes also contain a string of unique ORFs encoded on the same strand directly upstream of Tet P (Fig. 3a). Of particular interest is the presence of an ORF directly upstream of Tet P that encodes a putative large serine recombinase, similar to that found in the *P. sordellii* Tet P locus (Figs. 2a and 3a). An alignment was constructed between the Tet P locus from MD46 and the *P. sordellii* isolate W2945. While there were differences in the arrangement of the ORFs flanking Tet P between these two loci, significant levels of nucleotide identity were seen (Fig. 3a).

## Discussion

The variation in the chromosomal location and arrangement of Tet P between *C. difficile* and *P. sordellii* (Figs. 2a and 3a) suggests that a number of recombination events have taken place in the evolution of this element. Based on the homology of these sequences between *C. difficile* and *P. sordellii* these events may have occurred across species boundaries. Site-specific recombination events usually rely on or are marked by the presence of nucleotide repeats flanking the elements in question [30]. While no conserved direct or indirect repeat could be identified among the *P. sordellii* sequences, one repeat sequence was found to be present across the *C. difficile* genomes examined here. An 11 bp sequence was found on the genome of 08ACD0030/630 at the site where the unique regions flanking the Tet P loci are present (green lines, Fig. 3a). This sequence was also found directly flanking Tet P in both the porcine and human isolates, as well as flanking the string of ORFs upstream of Tet P in the human isolates (Fig. 3a). This repeat sequence may represent an insertion site for site specific recombination, being duplicated in the process in Tet P containing isolates, and these events may be catalyzed by the large serine recombinase associated with Tet P. These repeats may also serve as sites for RecA-mediated homologous recombination [31], which could also explain the insertion, deletion and rearrangement of genes at this locus. The direct repeat can also be found flanking Tet P within *P. sordellii* W2945 and provides a potential mechanism by which this element could have been excised from the chromosome prior to lateral gene transfer to another species. It should be noted that while not identical, the overall genomic context of the Tet P chromosomal loci appears to be conserved between *P. sordellii* and *C. difficile*, particularly in the region upstream of Tet P (Fig. 3b). This region of the chromosome may therefore represent a recombination hotspot within these species.

Although the genome sequences of the three porcine *C. difficile* isolates contain a Tet P locus, they were reported as being phenotypically sensitive to tetracycline [16]. We determined the MIC for tetracycline and minocycline for the two human Tet P^+^ *C. difficile* isolates, in comparison to Tet^R^ and Tet^S^ *C. difficile* controls (Table 1). The Clinical and Laboratory Standards Institute (CLSI) guidelines state that 16 mg/mL is the breakpoint MIC for tetracycline resistance in *C. difficile* [32], which renders isolates MCD43 and MCD46 as tetracycline sensitive (Table 1). However, the MIC values obtained for both tetracycline and minocycline for these isolates were substantially higher than those for the tetracycline sensitive control (Table 1), which may indicate that Tet P leads to an intermediate level of tetracycline resistance in *C. difficile*. While the clinical outcomes of such intermediate resistance in *C. difficile* are currently unknown, similar levels of resistance in other organisms, such as vancomycin intermediate-resistance in *Staphylococcus aureus*, [33, 34] have resulted in clinical challenges in the treatment of this organism. For *S. aureus*, both in vitro and clinical evidence indicates that isolates with intermediate-levels of vancomycin resistance may lead to treatment failure, potentially through the development of a higher MIC during treatment [33]. Members of the tetracycline family have been suggested for use in the prevention of *C. difficile* infection [35, 36]. However, our study and others have shown that determinants that provide full or intermediate resistance to tetracyclines are present in *C. difficile* strains [37], which should be considered or assessed before patient treatment commences.

## Conclusions

This study has demonstrated that ~ 20–30% of *P. sordellii* isolates tested in this study are resistant to the tetracycline family of antibiotics, including doxycycline, which has strong clinical relevance for *P. sordellii*-mediated intrauterine infections [9]. It should be noted that due to the rarity of *P. sordellii* infections the sample size analysed in this study is relatively small. The collection of further, more recent isolates is required to conclusively determine the levels of tetracycline resistance within *P. sordellii*. The determinant responsible for this resistance, Tet P, is present in highly variable genomic locations both within *P. sordellii* isolates and also among other clostridia. This variability in Tet P location and the potential association of this determinant with mobile elements in *P. sordellii* and *C. difficile*, and the location of Tet P on the conjugative plasmid pCW3 in *C. perfringens* [17, 18], suggests that Tet P has the capacity to spread rapidly among the clostridia. The dissemination of Tet P is not just a human clinical issue since we have shown that animal isolates of *P. sordellii* and *C. difficile* carry Tet P loci that are closely related to those of human isolates. Three related problems need to be understood when considering the dissemination of the Tet P determinant amongst clostridial strains: human health issues, animal health issues and the factor common to both of these problems, antibiotic use. The findings presented here reinforce the importance of a ‘one health’ approach in considering the use of antibiotics and the importance of genetically and phenotypically understanding the strains that carry resistance determinants.

## Methods

### Bacterial culture

Additional file 2: Table S2 lists the bacterial isolates used in this study. Unless stated otherwise, bacterial cultures were grown in HIS broth (37 g/L Heart Infusion (Oxoid), 5 g/L yeast extract, 1 g/L L-Cysteine, 0.375% (*w*/*v*) glucose) or on HIS agar (HIS broth with 15 g/L agar) at 37 °C in an anaerobic chamber (Coy Laboratory Products, Inc.) in an atmosphere of 10% H_2_, 10% CO_2_ and 80% N_2_. When required, antibiotics were included in the media at the following concentrations unless otherwise specified: tetracycline (Tet) 10 mg/L, streptomycin (Str) 200 mg/L, rifampicin (Rif) 25 mg/L and D-cycloserine (DCy) 250 mg/L.

### Minimum inhibitory concentration assays

To determine the tetracycline resistance profile of the *P. sordellii* isolates, the following assay, based on a previously published method [38], was performed. Note that *C. perfringens* isolates were included as controls. Isolates were grown at 37 °C overnight in 20 mL HIS broth with DCy under anaerobic conditions and the turbidity of the cultures determined by optical density at a wavelength of 600 nm (OD600). The cultures were diluted in 20 mL HIS broth with DCy to an OD600 of 0.1 and grown to a late-exponential growth phase, before dilution to an OD600 of 0.3 in fresh HIS broth with DCy. Duplicate 200 μl aliquots were added to 50 μl of HIS broth containing tetracycline (32 to 0.125 mg/L final volume), minocycline or doxycycline (12 to 0.047 mg/L final volume) or no antibiotic, each with an identical concentration of antibiotic solvent and DCy, within wells of a 96-well tissue culture plate. The trays were incubated at 37 °C for ~ 20 h under anaerobic conditions.

To determine a clinically relevant MIC for the *C. difficile* isolates, a broth microdilution method in standing with the CLSI guidelines and based on a previously published method [38], was used. An inoculum of 1-2 × 10^6^ cfu/ml from an actively growing HIS broth culture of *C. difficile* was diluted two fold in 96-well plates containing 100 μl of HIS broth containing tetracycline (32 to 0.125 mg/L final volume), minocycline or doxycycline (12 to 0.047 mg/L final volume) or no antibiotic, each with an identical concentration of antibiotic solvent. Plates were incubated at 37 °C for 24 h under anaerobic conditions. The MIC was determined to be the lowest concentration of the antibiotic for which no growth was observed.

### Molecular techniques

Genomic DNA extractions, PCR and restriction digestions were all performed as previously described [39]. Transformation of *C. perfringens* cells was carried out as previously described [40]. DNA for Southern hybridization was subjected to electrophoresis on a 0.8% Megabase agarose gel prior to transfer. Southern hybridization was carried out using *tetA*(P), *tetB*(P) and *sdl* specific DIG-labelled probes, as previously described [39]. Primers used for PCR in this study are listed in Additional file 8: Table S3.

### Bacterial matings

*P. sordellii* to *P. sordellii* and *P. sordellii* to *C. perfringens* matings were performed using mixed plate matings on solid media, as previously described for *C. perfringens* [41, 42].

### Genome sequence analysis

Sequence homology between loci was determined, and resulting graphics were produced, using EasyFig [43]. Multiple sequence alignments were conducted using Clustal Omega from EMBL-EBI tools [44]. Core SNP phylogeny, antibiotic resistance gene identification and pan genome analysis of *P. sordellii* sequencing data (see Additional file 2: Table S2 for accession numbers) was performed using the Nullarbor pipeline [45]. A contig containing Tet P was obtained from the draft genome sequence of *P. sordellii* W2922 produced as part of the Nullarbor pipeline [45]. This full contig was not present in the original draft genome of W2922 (CELK00000000) but is present when the original readset (ERR197363) from which the published assembly was derived is assembled with MegaHit version 1.1.3. This contig was therefore uploaded to the Third Party Annotation database under accession BK010701. Genome sequences not acquired as part of this study were obtained from GenBank [46], with the primary accession numbers for each sequence listed in Additional file 2: Table S2. *P. sordellii* contigs representing pCSTP1 from R28058 (CEKZ01000007) and pCSTP2 from SSCC37615 (CDNO01000006) were obtained from GenBank [46], and were trimmed to represent the closed plasmids. These plasmid sequences were re-annotated and deposited into the Third Party Annotation database under accession numbers BK010450 (pCSTC1) and BK010451 (pCSTC2).

*C. difficile* genomic DNA was sequenced using the Illumina NextSeq500 platform with 2×150 bp PE reads. Megahit v1.1.3 [47], as part of the Nullarbor pipeline [45], was used for contig assembly. Abricate v0.2 (Melbourne Bioinformatics) as part of the Nullarbor pipeline [45], was used for identifying Tet^R^ genes among the *C. difficile* draft genomes. Annotation of sequences was performed using Prokka [48], followed by further manual annotation using Artemis [49], based on sequence identity to characterised genes or hits in the Conserved Domain Database or the Phyre2 server [24, 25]. The contigs containing Tet P from *C. difficile* MCD43 (MH041492) and MCD46 (MH041493) were deposited via BankIt to GenBank [46].

## Additional files

Additional file 1: **Figure S1.** The Tet P tetracycline resistance determinant and surrounding regions in *C. perfringens*. Characterized regulatory elements upstream of the Tet P determinant are annotated and include the promoter P3 and the transcriptional terminator T1. (JPG 607 kb)

Additional file 2: **Table S2.** Bacterial isolates, genomic DNA sequences and sequencing data used in this study. (PDF 274 kb)

Additional file 3: **Table S1.** Matrices displaying the nucleotide identity (%) of the Tet P regulatory region comprising 650 bp directly upstream of the *tetA*(P) start codon, as well as the amino acid identity (%) of TetA(P) and TetB(P), from isolates of *P. sordellii* and *C. difficile* (*Cd*) compared with that of pCW3 from *C. perfringens* (*Cp*) CW92. (PDF 167 kb)

Additional file 4: **Figure S2.** Nucleotide alignment of the upstream regulatory regions of Tet P in *P. sordellii* and *C. difficile* isolates compared to that of *C. perfringens* pCW3 from strain CW92. The sequence from *P. sordellii* isolates that encode Tet P on small plasmids is represented by R28058 (but includes R15892, SSCC37615, R32977 and SSCC19939, Table S1). The sequence from *P. sordellii* isolates that appear to encode Tet P on the chromosome, with the exception of W2945, is represented by SSCC18392 (but includes SSCC32135, R32462, JGS6961, W3026, AM370 and W2922, Table S1). The beginning of the *tetA*(P) gene is colored red (red arrow) with the start codon in bold. The predicted ribosome binding sites (RBS) for these sequences are in black bold font. The region corresponding to the *C. perfringens* pCW3 Tet P promoter, P3, is highlighted in green with the predicted − 10 and − 35 boxes shown in bold. The inverted repeats that correspond to the T1 transcriptional terminator are highlighted in blue and indicated with inverted black arrows. (PDF 274 kb)

Additional file 5: **Figure S3.** Nucleotide alignment of the regions flanking *regA* in *P. sordellii* Tet P^+^ isolates. Sequences for the *regA*^+^ isolates SSCC32125, R32462, JGS6961 and SSCC18392 are compared to the *regA*^−^ isolate W2945. The direct repeats flanking *regA* are shown in bold red font and indicated by a black arrow beneath the sequence. The coding region of *regA* is colored purple. A gap in the alignment representing the majority of *regA* is indicated by three large dots. The start and stop codons of *regA* are in bold and annotated on the sequence. The *regA*^−^ isolates W3026, AM370 and W2922 have not been included in this alignment; however, a single site identical to the repeat is present for this isolate, equidistant to Tet P, as for W2945. (PDF 238 kb)

Additional file 6: **Figure S4.** Comparison of the genomic location of *tetA*(P) and *tetB*(P) among *P. sordellii* isolates using Southern hybridization analysis. Genomic DNA from *C. perfringens* isolate JIR56 was used as a *tetA/B*(P) positive control and *sdl* negative control, and genomic DNA from the *P. sordellii* isolates was used as *sdl* positive controls. *P. sordellii* isolate SSCC33587 was included as a Tet P negative control. Genomic DNA from *P. sordellii* isolates and *C. perfringens* strain JIR56 was either undigested (U) or digested with *Eco*RI (D). Blots were probed with DIG-labelled DNA either specific for *sdl* (chromosomal *P. sordellii* marker), *tetA*(P) or *tetB*(P). In the chromosomal Tet P isolates, both *tetA*(P) and *tetB*(P) probes hybridized to a band which represented undigested chromosomal DNA. This chromosomal location was confirmed using a *sdl* (chromosomal marker)-specific probe, which produced a fragment of a similar size to that seen with *tetA*(P) and *tetB*(P). For the undigested genomic DNA of the remaining 3 Tet^R^ isolates, distinct lower-molecular weight bands were observed for both the *tetA*(P) and *tetB*(P) blots. When digested with EcoRI and probed with *tetA*(P) or *tetB*(P), pCSTC1 and pCSTC2-carrying isolates produced bands of ~ 8.5 kb and ~ 9.8 kb, respectively, which are the expected sizes of these plasmids. (JPG 2793 kb)

Additional file 7: **Figure S5.** PCR analysis of *C. perfringens* JIR325 transformed with *P. sordellii* genomic DNA (gDNA) from isolates SSCC37615 or SSCC18838. Three separate isolates from each transformation were analysed (Lanes 1–6). PCR reactions were subjected to agarose gel electrophoresis against a Hyperladder 1 kb marker (Bioline), with relevant sizes indicated in bp. (a) PCR to detect the Tet P determinant using the internal *tetA*(P) primer DLP104 and the internal *tetB*(P) primer DLP105. A product of the expected size was observed for all reactions with the exception of non-transformed JIR325 (negative control) and the no DNA control. (b) PCR to detect the *C. perfringens* chromosomal gene *plc* using internal primers JRP2873 and JRP2874. A product of the expected size was observed for all reactions with the exception of *P. sordellii* (*Ps*) isolate R28058 (negative control) and the no DNA control. (JPG 58 kb)

Additional file 8: **Table S3.** Oligonucleotide primers used in PCR. (+) forward primer, (−) reverse primer. (PDF 89 kb)

## Abbreviations

CLSI: Clinical and Laboratory Standards Institute
MIC: minimum inhibitory concentration
PCR: polymerase chain reaction
RPP: ribosomal protection protein
Tet^R^: tetracycline resistant
Tet^S^: tetracycline sensitive
