# Supplementary figures and images for: Paeniclostridium sordellii and Clostridioides difficile encode similar and clinically relevant tetracycline resistance loci in diverse genomic locations
Source: BMC Microbiol. 2019 Mar 4;19:53. doi: 10.1186/s12866-019-1427-5 (PMC6399922; doi:10.1186/s12866-019-1427-5)

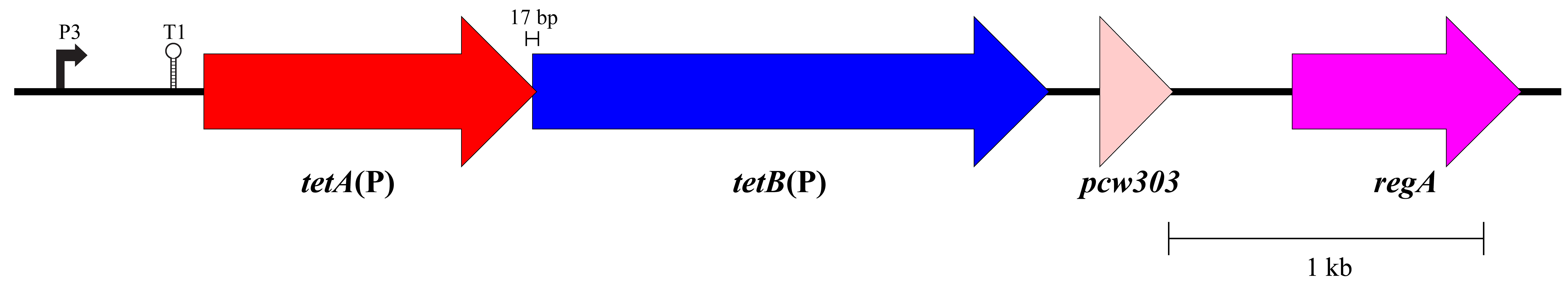

Supplement: Supplementary file 1 — Figure S1. The Tet P tetracycline resistance determinant and surrounding regions in C. perfringens. Characterized regulatory elements upstream of the Tet P determinant are annotated and include the promoter P3 and the transcriptional terminator T1. (JPG 607 kb) [file 12866_2019_1427_MOESM1_ESM.jpg]

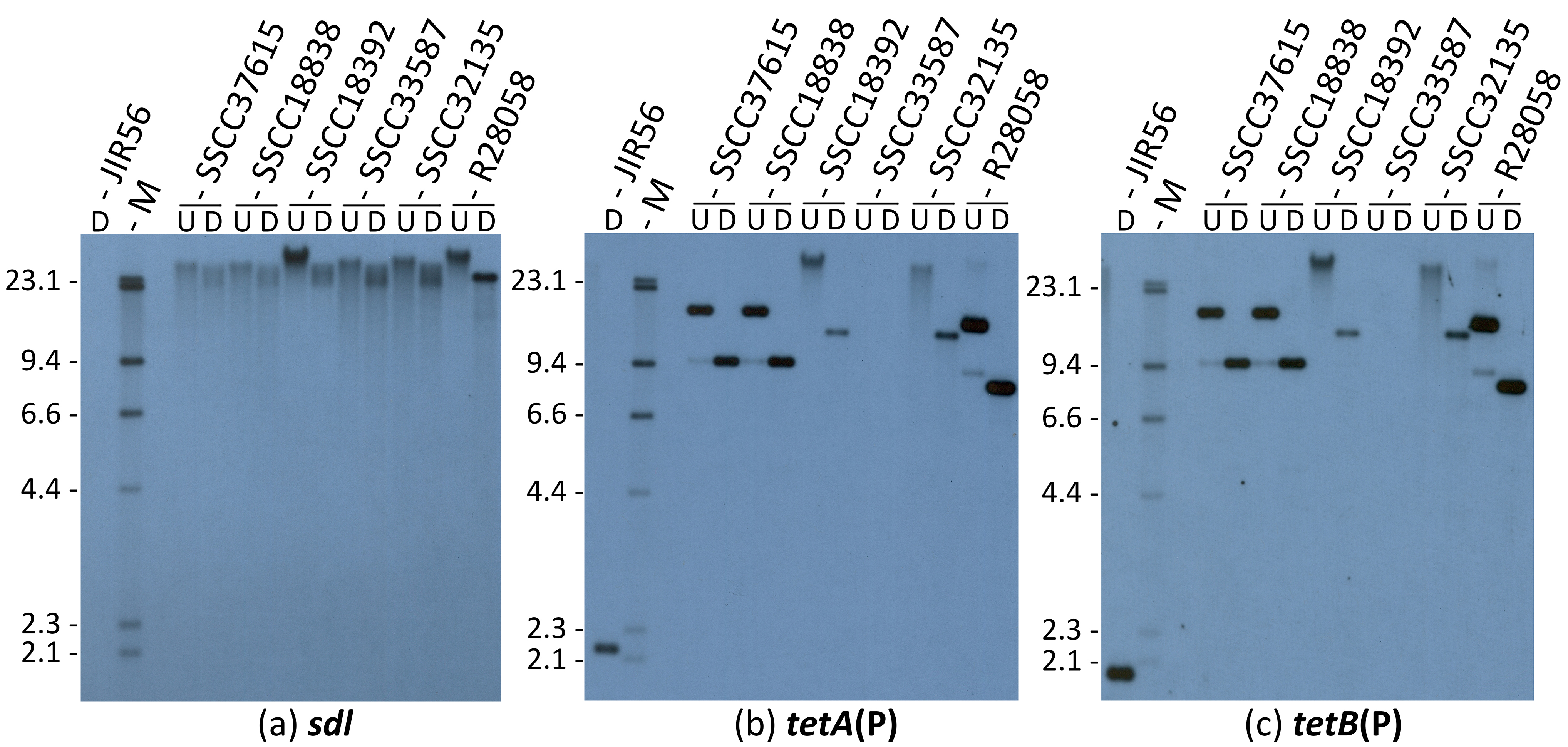

Supplement: Supplementary file 6 — Figure S4. Comparison of the genomic location of tetA(P) and tetB(P) among P. sordellii isolates using Southern hybridization analysis. Genomic DNA from C. perfringens isolate JIR56 was used as a tetA/B(P) positive control and sdl negative control, and genomic DNA from the P. sordellii isolates was used as sdl positive controls. P. sordellii isolate SSCC33587 was included as a Tet P negative control. Genomic DNA from P. sordellii isolates and C. perfringens strain JIR56 was either undigested (U) or digested with EcoRI (D). Blots were probed with DIG-labelled DNA either specific for sdl (chromosomal P. sordellii marker), tetA(P) or tetB(P). In the chromosomal Tet P isolates, both tetA(P) and tetB(P) probes hybridized to a band which represented undigested chromosomal DNA. This chromosomal location was confirmed using a sdl (chromosomal marker)-specific probe, which produced a fragment of a similar size to that seen with tetA(P) and tetB(P). For the undigested genomic DNA of the remaining 3 TetR isolates, distinct lower-molecular weight bands were observed for both the tetA(P) and tetB(P) blots. When digested with EcoRI and probed with tetA(P) or tetB(P), pCSTC1 and pCSTC2-carrying isolates produced bands of ~ 8.5 kb and ~ 9.8 kb, respectively, which are the expected sizes of these plasmids. (JPG 2793 kb) [file 12866_2019_1427_MOESM6_ESM.jpg]

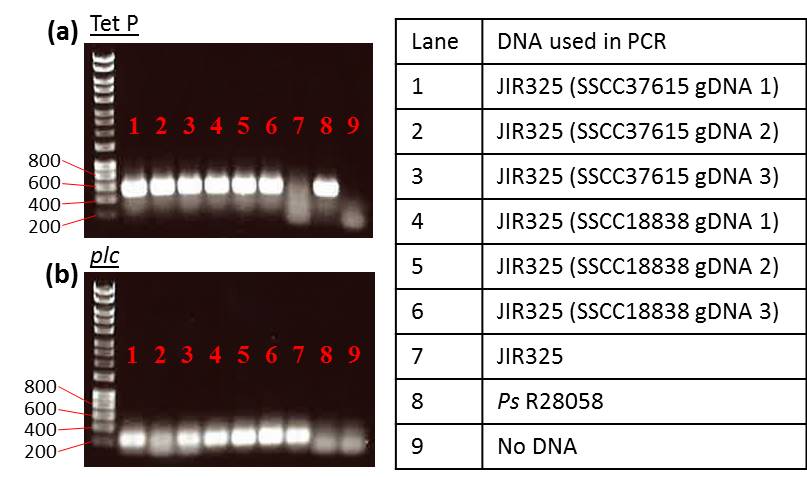

Supplement: Supplementary file 7 — Figure S5. PCR analysis of C. perfringens JIR325 transformed with P. sordellii genomic DNA (gDNA) from isolates SSCC37615 or SSCC18838. Three separate isolates from each transformation were analysed (Lanes 1–6). PCR reactions were subjected to agarose gel electrophoresis against a Hyperladder 1 kb marker (Bioline), with relevant sizes indicated in bp. (a) PCR to detect the Tet P determinant using the internal tetA(P) primer DLP104 and the internal tetB(P) primer DLP105. A product of the expected size was observed for all reactions with the exception of non-transformed JIR325 (negative control) and the no DNA control. (b) PCR to detect the C. perfringens chromosomal gene plc using internal primers JRP2873 and JRP2874. A product of the expected size was observed for all reactions with the exception of P. sordellii (Ps) isolate R28058 (negative control) and the no DNA control. (JPG 58 kb) [file 12866_2019_1427_MOESM7_ESM.jpg]
