# Supplementary material for: Paeniclostridium sordellii and Clostridioides difficile encode similar and clinically relevant tetracycline resistance loci in diverse genomic locations
Source: BMC Microbiol. 2019 Mar 4;19:53. doi: 10.1186/s12866-019-1427-5 (PMC6399922; doi:10.1186/s12866-019-1427-5)
Supplement: Supplementary file 2 — Table S2. Bacterial isolates, genomic DNA sequences and sequencing data used in this study. (PDF 274 kb) [file 12866_2019_1427_MOESM2_ESM.pdf]

Isolate	Characteristics	Tet P	MIC	Alignment sequence accession no.	Sequencing data accession no.
P. sordellii					
AM370	Unknown isolate	✓		CP014150	SRR3109670
ATCC 9714 [1, 2]	Reference, human, oedema		✓	LN679998	ERR197376
DA108 [2, 3]	Human, non-fatal endometriosis		✓	N/A	ERR197377
E204 [2]	Human, clinical isolate		✓	N/A	ERR197393
JGS444 [2]	Bovine, myonecrosis		✓	N/A	ERR197398
JGS445 [2]	Bovine, myonecrosis		✓	N/A	ERR197372
JGS6364 [2]	Bovine, myonecrosis		✓	N/A	ERR197397
JGS6382 [2, 4]	Bovine, myonecrosis		✓	N/A	ERR197373
JGS6956 [2]	Veterinary isolate			N/A	ERR197374
JGS6961 [2]	Veterinary isolate	✓		CDNI01000025	ERR197375
R15892 [2]	Human, clinical isolate	✓	✓	CEKU01000007	ERR197394
R26833 [2]	Human, blood isolate from diabetic			N/A	ERR197360
R27882 [2]	Human, knee amputation			N/A	ERR197359
R28058 [2]	Human, crushed hand	✓	✓	BK010450	ERR197358
R29426 [2]	Human, sudden death			N/A	ERR197357
R30684 [2]	Human, calf abscess			N/A	ERR197356
R31809 [2]	Human, knee injury			N/A	ERR197355
R32462 [2]	Human, endo-cervical discharge	✓		CEMX01000019	ERR197354
R32668 [2]	Human, wound			N/A	ERR197353
R32921 [2]	Human, death during pregnancy			N/A	ERR197352
R32977 [2]	Human, knee replacement	✓		CELC01000006	ERR197351
SSCC18392 [2]	Human, blood isolate	✓	✓	CDNE01000023	ERR197383
SSCC18838 [2]	Human, blood isolate	✓	✓	CDNK01000006	ERR197382
SSCC26591 [2]	Human, blood isolate		✓	N/A	ERR197380
SSCC32135 [2]	Human, blood isolate	✓	✓	CDNQ01000027	ERR197386
SSCC33587 [2]	Human, blood isolate		✓	N/A	ERR197385

SSCC33589 [2]	Human, blood isolate		✓	N/A	ERR197378
SSCC35109 [2]	Human, blood isolate		✓	N/A	ERR197384
SSCC37615 [2]	Human, blood isolate	✓	✓	BK010451	ERR197381
SSCC42239 [2]	Human, blood isolate		✓	N/A	ERR197379
UMC1 [2, 5]	Human, allograft isolate		✓	N/A	ERR197387
UMC2 [2, 5]	Human, allograft isolate		✓	N/A	ERR197388
W10 [2]	Veterinary isolate			N/A	ERR197362
UMC164 [2, 5]	Human, allograft isolate		✓	N/A	ERR197389
UMC178 [2, 5]	Human, allograft isolate		✓	N/A	ERR197390
UMC4401 [2, 5]	Human, allograft isolate		✓	N/A	ERR197391
UMC4404 [2, 5]	Human, allograft isolate		✓	N/A	ERR197392
W2922 [2]	Veterinary isolate	✓		CELK01000144, BK010701	ERR197363
W2945 [2]	Veterinary isolate	✓		CELI01000026	ERR197364
W2946 [2]	Veterinary isolate	✓		N/A	ERR197365*
W2947 [2]	Veterinary isolate			N/A	ERR197366*
W2948 [2]	Veterinary isolate			N/A	ERR197367
W2967 [2]	Veterinary isolate			N/A	ERR197361
W2975 [2]	Veterinary isolate			N/A	ERR197368
W3025 [2]	Veterinary isolate			N/A	ERR197369
W3026 [2]	Veterinary isolate	✓		CELD01000042	ERR197370
C. perfringens					
Strain 13 [6]	Reference, Tet^S		✓	N/A	N/A
JIR4 [7]	Strain 13 background, contains pCW3, Tet^R	✓	✓	N/A	N/A
CW92 [7, 8]	WT isolate, contains pCW3	✓		DQ366035	N/A
C. difficile					
Strain 630 [9, 10]	Human, pseudomembranous colitis, Tet^R		✓	CP010905	N/A
CD37 [11]	Clinical isolate, Tet^S		✓	N/A	N/A
MCD43	Human, clinical isolate	✓	✓	MH041492	N/A
MCD46	Human, clinical isolate	✓	✓	MH041493	N/A
P12 [12]	Porcine isolate	✓		ERS1078747	N/A
08ACD0030	Unknown			CP010888	N/A

* Sequencing data indicates mixed isolates

Strain/Sequence References

1. Hall IC, Scott, J.P.: ***Bacillus sordellii*, a Cause of Malignant Edema in Man**. *J Infect Dis* 1927, **41**:329-335.
2. Couchman EC, Browne HP, Dunn M, Lawley TD, Songer JG, Hall V, Petrovska L, Vidor C, Awad M, Lyras D *et al*: ***Clostridium sordellii* genome analysis reveals plasmid localized toxin genes encoded within pathogenicity loci**. *BMC Genomics* 2015, **16**(1):392.
3. Hao Y, Senn T, Opp JS, Young VB, Thiele T, Srinivas G, Huang SK, Aronoff DM: **Lethal toxin is a critical determinant of rapid mortality in rodent models of *Clostridium sordellii* endometritis**. *Anaerobe* 2010, **16**(2):155-160.
4. Walk ST, Jain R, Trivedi I, Grossman S, Newton DW, Thelen T, Hao Y, Songer JG, Carter GP, Lyras D *et al*: **Non-toxigenic *Clostridium sordellii*: Clinical and microbiological features of a case of cholangitis-associated bacteremia**. *Anaerobe* 2011, **17**(5):252-256.
5. Voth DE, Martinez OV, Ballard JD: **Variations in lethal toxin and cholesterol-dependent cytolysin production correspond to differences in cytotoxicity among strains of *Clostridium sordellii***. *FEMS Microbiol Lett* 2006, **259**(2):295-302.
6. Lyristis M, Bryant AE, Sloan J, Awad MM, Nisbet IT, Stevens DL, Rood JI: **Identification and molecular analysis of a locus that regulates extracellular toxin production in *Clostridium perfringens***. *Mol Microbiol* 1994, **12**(5):761-777.
7. Rood JI: **Transferable tetracycline resistance in *Clostridium perfringens* strains of porcine origin**. *Can J Microbiol* 1983, **29**(10):1241-1246.
8. Bannam TL, Teng WL, Bulach D, Lyras D, Rood JI: **Functional identification of conjugation and replication regions of the tetracycline resistance plasmid pCW3 from *Clostridium perfringens***. *J Bacteriol* 2006, **188**(13):4942-4951.
9. Wüst J, Sullivan NM, Hardegger U, Wilkins TD: **Investigation of an outbreak of antibiotic-associated colitis by various typing methods**. *J Clin Microbiol* 1982, **16**(6):1096-1101.
10. Riedel T, Bunk B, Thürmer A, Spröer C, Brzuszkiewicz E, Abt B, Gronow S, Liesegang H, Daniel R, Overmann J: **Genome Resequencing of the Virulent and Multidrug-Resistant Reference Strain *Clostridium difficile* 630**. *Genome Announcements* 2015, **3**(2).
11. Smith CJ, Markowitz SM, Macrina FL: **Transferable tetracycline resistance in Clostridium difficile**. *Antimicrob Agents Chemother* 1981, **19**(6):997-1003.
12. Knight DR, Squire MM, Collins DA, Riley TV: **Genome Analysis of *Clostridium difficile* PCR Ribotype 014 Lineage in Australian Pigs and Humans Reveals a Diverse Genetic Repertoire and Signatures of Long-Range Interspecies Transmission**. *Front Microbiol* 2017, **7**(2138).
