# Supplementary material for: Paeniclostridium sordellii and Clostridioides difficile encode similar and clinically relevant tetracycline resistance loci in diverse genomic locations
Source: BMC Microbiol. 2019 Mar 4;19:53. doi: 10.1186/s12866-019-1427-5 (PMC6399922; doi:10.1186/s12866-019-1427-5)
Supplement: Supplementary file 3 — Table S1. Matrices displaying the nucleotide identity (%) of the Tet P regulatory region comprising 650 bp directly upstream of the tetA(P) start codon, as well as the amino acid identity (%) of TetA(P) and TetB(P), from isolates of P. sordellii and C. difficile (Cd) compared with that of pCW3 from C. perfringens (Cp) CW92. (PDF 167 kb) [file 12866_2019_1427_MOESM3_ESM.pdf]

**Upstream Tet P**	1	2	3	4	5	6	7	8	9	10	11	12	13	14	15	16
1: *Cp* CW92	100.0	89.1	89.1	89.1	89.1	89.1	88.3	88.3	89.4	89.4	89.4	89.4	89.4	89.4	89.4	89.5
2: R28058	89.1	100.0	100.0	100.0	100.0	100.0	88.6	88.6	89.7	89.7	89.7	89.7	89.7	89.7	89.7	89.8
3: SSCC37615	89.1	100.0	100.0	100.0	100.0	100.0	88.6	88.6	89.7	89.7	89.7	89.7	89.7	89.7	89.7	89.8
4: R15892	89.1	100.0	100.0	100.0	100.0	100.0	88.6	88.6	89.7	89.7	89.7	89.7	89.7	89.7	89.7	89.8
5: R32977	89.1	100.0	100.0	100.0	100.0	100.0	88.6	88.6	89.7	89.7	89.7	89.7	89.7	89.7	89.7	89.8
6: SSCC18838	89.1	100.0	100.0	100.0	100.0	100.0	88.6	88.6	89.7	89.7	89.7	89.7	89.7	89.7	89.7	89.8
7: W2945	88.3	88.6	88.6	88.6	88.6	88.6	100.0	100.0	99.7	99.7	99.7	99.7	99.7	99.7	99.7	99.5
8: *Cd* MCD46	88.3	88.6	88.6	88.6	88.6	88.6	100.0	100.0	99.7	99.7	99.7	99.7	99.7	99.7	99.7	99.5
9: R32462	89.4	89.7	89.7	89.7	89.7	89.7	99.7	99.7	100.0	100.0	100.0	100.0	100.0	100.0	100.0	99.8
10: SSCC18392	89.4	89.7	89.7	89.7	89.7	89.7	99.7	99.7	100.0	100.0	100.0	100.0	100.0	100.0	100.0	99.8
11: SSCC32135	89.4	89.7	89.7	89.7	89.7	89.7	99.7	99.7	100.0	100.0	100.0	100.0	100.0	100.0	100.0	99.8
12: W3026	89.4	89.7	89.7	89.7	89.7	89.7	99.7	99.7	100.0	100.0	100.0	100.0	100.0	100.0	100.0	99.8
13: JGS6961	89.4	89.7	89.7	89.7	89.7	89.7	99.7	99.7	100.0	100.0	100.0	100.0	100.0	100.0	100.0	99.8
14: AM370	89.4	89.7	89.7	89.7	89.7	89.7	99.7	99.7	100.0	100.0	100.0	100.0	100.0	100.0	100.0	99.8
15: W2922	89.4	89.7	89.7	89.7	89.7	89.7	99.7	99.7	100.0	100.0	100.0	100.0	100.0	100.0	100.0	99.8
16: *Cd* P12	89.5	89.8	89.8	89.8	89.8	89.8	99.5	99.5	99.8	99.8	99.8	99.8	99.8	99.8	99.8	100.0

**TetA(P)**	1	2	3	4	5	6	7	8	9	10	11	12	13	14	15	16
1: *Cp* CW92	100.0	89.5	89.5	89.5	89.5	89.5	88.3	88.3	88.3	88.3	88.3	88.3	88.3	88.3	88.3	88.3
2: R28058	89.5	100.0	100.0	100.0	100.0	100.0	96.4	96.4	96.4	96.4	96.4	96.4	96.4	96.4	96.4	96.4
3: SSCC37615	89.5	100.0	100.0	100.0	100.0	100.0	96.4	96.4	96.4	96.4	96.4	96.4	96.4	96.4	96.4	96.4
4: R15892	89.5	100.0	100.0	100.0	100.0	100.0	96.4	96.4	96.4	96.4	96.4	96.4	96.4	96.4	96.4	96.4
5: R32977	89.5	100.0	100.0	100.0	100.0	100.0	96.4	96.4	96.4	96.4	96.4	96.4	96.4	96.4	96.4	96.4
6: SSCC18838	89.5	100.0	100.0	100.0	100.0	100.0	96.4	96.4	96.4	96.4	96.4	96.4	96.4	96.4	96.4	96.4
7: R32462	88.3	96.4	96.4	96.4	96.4	96.4	100.0	100.0	100.0	100.0	100.0	100.0	100.0	100.0	100.0	100.0
8: SSCC18392	88.3	96.4	96.4	96.4	96.4	96.4	100.0	100.0	100.0	100.0	100.0	100.0	100.0	100.0	100.0	100.0
9: SSCC32135	88.3	96.4	96.4	96.4	96.4	96.4	100.0	100.0	100.0	100.0	100.0	100.0	100.0	100.0	100.0	100.0
10: W2945	88.3	96.4	96.4	96.4	96.4	96.4	100.0	100.0	100.0	100.0	100.0	100.0	100.0	100.0	100.0	100.0
11: W3026	88.3	96.4	96.4	96.4	96.4	96.4	100.0	100.0	100.0	100.0	100.0	100.0	100.0	100.0	100.0	100.0
12: JGS6961	88.3	96.4	96.4	96.4	96.4	96.4	100.0	100.0	100.0	100.0	100.0	100.0	100.0	100.0	100.0	100.0
13: AM370	88.3	96.4	96.4	96.4	96.4	96.4	100.0	100.0	100.0	100.0	100.0	100.0	100.0	100.0	100.0	100.0
14: W2922	88.3	96.4	96.4	96.4	96.4	96.4	100.0	100.0	100.0	100.0	100.0	100.0	100.0	100.0	100.0	100.0
15: *Cd* P12	88.3	96.4	96.4	96.4	96.4	96.4	100.0	100.0	100.0	100.0	100.0	100.0	100.0	100.0	100.0	100.0
16: *Cd* MCD46	88.3	96.4	96.4	96.4	96.4	96.4	100.0	100.0	100.0	100.0	100.0	100.0	100.0	100.0	100.0	100.0

**TetB(P)**	1	2	3	4	5	6	7	8	9	10	11	12	13	14	15	16
1: R28058	100.0	100.0	100.0	100.0	100.0	96.9	96.9	96.9	96.9	96.9	96.9	96.9	96.9	96.9	96.9	96.5
2: SSCC37615	100.0	100.0	100.0	100.0	100.0	96.9	96.9	96.9	96.9	96.9	96.9	96.9	96.9	96.9	96.9	96.5
3: R15892	100.0	100.0	100.0	100.0	100.0	96.9	96.9	96.9	96.9	96.9	96.9	96.9	96.9	96.9	96.9	96.5
4: R32977	100.0	100.0	100.0	100.0	100.0	96.9	96.9	96.9	96.9	96.9	96.9	96.9	96.9	96.9	96.9	96.5
5: SSCC18838	100.0	100.0	100.0	100.0	100.0	96.9	96.9	96.9	96.9	96.9	96.9	96.9	96.9	96.9	96.9	96.5
6: R32462	96.9	96.9	96.9	96.9	96.9	100.0	100.0	100.0	100.0	100.0	100.0	100.0	100.0	100.0	100.0	97.9
7: SSCC18392	96.9	96.9	96.9	96.9	96.9	100.0	100.0	100.0	100.0	100.0	100.0	100.0	100.0	100.0	100.0	97.9
8: SSCC32135	96.9	96.9	96.9	96.9	96.9	100.0	100.0	100.0	100.0	100.0	100.0	100.0	100.0	100.0	100.0	97.9
9: W2945	96.9	96.9	96.9	96.9	96.9	100.0	100.0	100.0	100.0	100.0	100.0	100.0	100.0	100.0	100.0	97.9
10: W3026	96.9	96.9	96.9	96.9	96.9	100.0	100.0	100.0	100.0	100.0	100.0	100.0	100.0	100.0	100.0	97.9
11: JGS6961	96.9	96.9	96.9	96.9	96.9	100.0	100.0	100.0	100.0	100.0	100.0	100.0	100.0	100.0	100.0	97.9
12: AM370	96.9	96.9	96.9	96.9	96.9	100.0	100.0	100.0	100.0	100.0	100.0	100.0	100.0	100.0	100.0	97.9
13: W2922	96.9	96.9	96.9	96.9	96.9	100.0	100.0	100.0	100.0	100.0	100.0	100.0	100.0	100.0	100.0	97.9
14: *Cd* P12	96.9	96.9	96.9	96.9	96.9	100.0	100.0	100.0	100.0	100.0	100.0	100.0	100.0	100.0	100.0	97.9
15: *Cd* MCD46	96.9	96.9	96.9	96.9	96.9	100.0	100.0	100.0	100.0	100.0	100.0	100.0	100.0	100.0	100.0	97.9
16: *Cp* CW92	96.5	96.5	96.5	96.5	96.5	97.9	97.9	97.9	97.9	97.9	97.9	97.9	97.9	97.9	97.9	100.0
