# Supplementary material for: Paeniclostridium sordellii and Clostridioides difficile encode similar and clinically relevant tetracycline resistance loci in diverse genomic locations
Source: BMC Microbiol. 2019 Mar 4;19:53. doi: 10.1186/s12866-019-1427-5 (PMC6399922; doi:10.1186/s12866-019-1427-5)
Supplement: Supplementary file 4 — Figure S2. Nucleotide alignment of the upstream regulatory regions of Tet P in P. sordellii and C. difficile isolates compared to that of C. perfringens pCW3 from strain CW92. The sequence from P. sordellii isolates that encode Tet P on small plasmids is represented by R28058 (but includes R15892, SSCC37615, R32977 and SSCC19939, Table S1). The sequence from P. sordellii isolates that appear to encode Tet P on the chromosome, with the exception of W2945, is represented by SSCC18392 (but includes SSCC32135, R32462, JGS6961, W3026, AM370 and W2922, Table S1). The beginning of the tetA(P) gene is colored red (red arrow) with the start codon in bold. The predicted ribosome binding sites (RBS) for these sequences are in black bold font. The region corresponding to the C. perfringens pCW3 Tet P promoter, P3, is highlighted in green with the predicted − 10 and − 35 boxes shown in bold. The inverted repeats that correspond to the T1 transcriptional terminator are highlighted in blue and indicated with inverted black arrows. (PDF 274 kb) [file 12866_2019_1427_MOESM4_ESM.pdf]

```
Cp CW92           TAAAAAAGACTCTATAATTTAGAGTCTTTTTTGATTTTAATTAAATTTGGATAAATATAA 60
R28058            CGTTTTAATGTATTC-----GTTTCCAAATATAATAATAGTAATAAGAAAATAAATATAA 55
W2945             CTTTTGTGTATATAAGTTATTTGAAGTAATAAAGTTTCAATTGAATTTGGATAAATATGA 60
Cd MCD46          CTTTTGTGTATATAAGTTATTTGAAGTAATAAAGTTTCAATTGAATTTGGATAAATATGA 60
SSCC18392         -----GTGTATATAAGTTATTTGAAGTAATAAAGTTTCAATTGAATTTGGATAAATATGA 55
Cd P12            -----GTGTATATAAGTTATTTGAAGTAATAAAGTTTCAATTGAATTTGGATAAATATAA 55
                            * *                *    *   * *   *     ******** *

                                    Promoter P3
                              -35                    -10
Cp CW92           ATATAAAAATATTGAAAAAAAATAAAAAAAAAATATATAATGTAGTTATGCTAAAATATAA 120
R28058            AAGTAAAAGCATTGAAGAAAATTAAAAATTTATATATAATGTAATTATACTAAAATATAA 115
W2945             ATATAAAAGTATTGAAAA-AAATAAAAGTTTATATATAATGTAATTATACTAAAATATAA 119
Cd MCD46          ATATAAAAGTATTGAAAA-AAATAAAAGTTTATATATAATGTAATTATACTAAAATATAA 119
SSCC18392         ATATAAAAGTATTGAAAAAAAATAAAAGTTTATATATAATGTAATTATACTAAAATATAA 115
Cd P12            ATATAAAAGTATTGAAAAAAAATAAAAGTTTATATATAATGTAATTATACTAAAATATAA 115
                  *  *****  ****** * ** *****    ************ **** ***********

Cp CW92           ACATGAAAAATATAAATGTAAAGGTGTTGTAAATATGCGCAAAGAGGTAATTTTTAATTA 180
R28058            ACATGAAAAATATAAATGTAAAGGGGTTATAAATATGCGCAAAGAGGTAGTTTTTAACTA 175
W2945             ACATGAAAAATATAAATGTAAAGGAGATGTAAATATGCGCAAAGAGGTAGTTTTTAACTA 179
Cd MCD46          ACATGAAAAATATAAATGTAAAGGAGATGTAAATATGCGCAAAGAGGTAGTTTTTAACTA 179
SSCC18392         ACATGAAAAATATAAATGTAAAGGAGATGTAAATATGCGCAAAGAGGTAGTTTTTAACTA 175
Cd P12            ACATGAAAAATATAAATGTAAAGGAGATGTAAATATGCGCAAAGAGGTAGTTTTTAACTA 175
                  ************************ * * ******************** ******* **

Cp CW92           AATTAACTGAATAAGGATTATTTTTAAAAGTATATGAA--AAATCTTGAATAACTCAAGG 238
R28058            AACTAACTGAATAAGGATTATTTTAAAAAGTATATGAAA-AAATCTTGAATAACTCAAGG 234
W2945             AACTAACTGAATAAGGATTATTTTAAAAAGTATATGAAAAAAATCTTGGATAACTCAAGG 239
Cd MCD46          AACTAACTGAATAAGGATTATTTTAAAAAGTATATGAAAAAAATCTTGGATAACTCAAGG 239
SSCC18392         AACTAACTGAATAAGGATTATTTTAAAAAGTATATGAAAAAAATCTTGGATAACTCAAGG 235
Cd P12            AACTAACTGAATAAGGATTATTTTAAAAAGTATATGAAAAAAATCTTGGATAACTCAAGG 235
                  ** ********************* *************  ******** ***********

Cp CW92           TTTATTTGTGGTGGAATCAATTATTTATAATATTGATATTACCATGTTTATTTGTGTACT 298
R28058            TTTATTTGTGGTAGAATCAATTATTGATAATATTGATATTACCATGTTTATTTGTGTACT 294
W2945             TTTATTTGTGGTAGAAACAATTATTAATAATATTGATATTACCATGTCTATTTGTGTACT 299
Cd MCD46          TTTATTTGTGGTAGAAACAATTATTAATAATATTGATATTACCATGTCTATTTGTGTACT 299
SSCC18392         TTTATTTGTGGTAGAAACAATTATTAATAATATTGATATTACCATGTCTATTTGTGTACT 295
Cd P12            TTTATTTGTGGTAGAAACAATTATTAATAATATTGATATTACCATGTCTATTTGTGTACT 295
                  ************ *** ******** ********************* ************

Cp CW92           CTAAAAATAATCAGTATTATAATGAGCATGTTACATCTTTATTTGCAGGATAGGAAAACC 358
R28058            CTAAAAATAATCAGTATTATAATGAGCATGTTACACCTCTATTTGCAGAATGGGAAAACT 354
W2945             CTAAAAATAATCAGTATTATAATGAGCATATTACATCTTTATTTGAAGAATAGGAAAACT 359
Cd MCD46          CTAAAAATAATCAGTATTATAATGAGCATATTACATCTTTATTTGAAGAATAGGAAAACT 359
SSCC18392         CTAAAAATAATCAGTATTATAATGAGCATATTACATCTTTATTTGAAGAATAGGAAAACT 355
Cd P12            CTAAAAATAATCAGTATTATAATGAGCATATTACATCTTTATTTGAAGAATAGGAAAACT 355
                  ***************************** ***** ** ****** ** ** *******

Cp CW92           GTGTTCTAAACACGGTATTTTTATGTCTATTTTTGCTATATA--TAGATATGGTATAAGA 416
R28058            GTGTTCTAAACACAGTATTTTTATGTCTATTTTTGCTACATTTATAGATATGGTATAAGA 414
W2945             GTGTTCTAAACACGGTATTTTTATGCCTATTTTTACTATATTTATAGATATGATATAAGA 419
Cd MCD46          GTGTTCTAAACACGGTATTTTTATGCCTATTTTTACTATATTTATAGATATGATATAAGA 419
SSCC18392         GTGTTCTAAACACGGTATTTTTATGCCTATTTTTACTATATTTATAGATATGATATAAGA 415
Cd P12            GTGTTCTAAACACGGTATTTTTATGCCTATTTTTACTATATTTATAGATATGATATAAGA 415
                  ************* *********** ******** *** **   ******** *******
```

```
Cp CW92        TTTTAATTTGAATAGCTTAATCATAAGTTATATA-ATTAAAGTTCTATATTTTATGTTTA  475
R28058         TTTTAATTTGAATAGCTTAATAATAAGCTATATAAATTAAAGTTCTATATTTCATGTTTA  474
W2945          CTTTAATTTGCATAGCTTAATCATAAGTTATATAAATTAAAGTTGTATATTTCATGTTTA  479
Cd MCD46       CTTTAATTTGCATAGCTTAATCATAAGTTATATAAATTAAAGTTGTATATTTCATGTTTA  479
SSCC18392      CTTTAATTTGCATAGCTTAATCATAAGTTATATAAATTAAAGTTGTATATTTCATGTTTA  475
Cd P12         CTTTAATTTGCATAGCTTAATCATAAGTTATATAAATTAAAGTTGTATATTTCATGTTTA  475
                ********* ********** ***** ****** ********* ******* *******

Cp CW92        TATTTAGGTTATATGATTATGAGGCTGTAAGTGAATTATGTTCATTTACAGCTTTT-TTT  534
R28058         TATTTAGGTTATATAATTATGAGGCTGTAAGTGAATTATATTCATTTACAGCCTTTTTTT  534
W2945          TATTTAGGTTATATAATTT---------------------------------------TT  500
Cd MCD46       TATTTAGGTTATATAATTT---------------------------------------TT  500
SSCC18392      TATTTAGGTTATATAATTATGAGGCTGTAAGTGAATTATGTTCACTTACAGCTTTT-TTT  534
Cd P12         TATTTAGGTTATATAATTATGAGGCTGTAAGTGAATTATGTTCACTTACAGCTTTT-TTT  534
               ************** ***                                        **
                                     Transcriptional terminator T1

Cp CW92        GTTTAAGGAGATTATTTTGATGATTTATATATTTAAATATGAAGATTATGAAAAACAAAC  594
R28058         GTTTAAGGAGATTATTTTGATGATTTATATATTTAAATATGAAGGTTATGAAAAACAAAC  594
W2945          ATTTAAGGAGATTATTTTGATGATTTATATATTTAAATATGAAGATTATGAAAAACAAAT  560
Cd MCD46       ATTTAAGGAGATTATTTTGATGATTTATATATTTAAATATGAAGATTATGAAAAACAAAT  560
SSCC18392      ATTTAAGGAGATTATTTTGATGATTTATATATTTAAATATGAAGATTATGAAAAACAAAT  594
Cd P12         ATTTAAGGAGATTATTTTGATGATTTATATATTTAAATATGAAGATTATGAAAAACAAAT  594
                ******************************************* *************

                                   RBS
Cp CW92        AAATTTTTTAAGAAATACTAGGATGGTGATGATTAATGGTTAATAAACTT    644
R28058         AAATTTTTTAAGAAATACTAGGATGGTGATGATTAATGGTTAATAAACTT    644
W2945          AAATTTTTTTAAAAATACTAGGATGGTGATTATTAATGGTTAATAAACTT    610
Cd MCD46       AAATTTTTTTAAAAATACTAGGATGGTGATTATTAATGGTTAATAAACTT    610
SSCC18392      AAATTTTTTTAAAAATACTAGGATGGTGATTATTAATGGTTAATAAACTT    644
Cd P12         AAATTTTTTTAAAAATACTAGGATGGTGATTATTAATGGTTAATAAACTT    644
               ********* * ****************** ******************
                                                      tetA(P)
```
