# Supplementary material for: Paeniclostridium sordellii and Clostridioides difficile encode similar and clinically relevant tetracycline resistance loci in diverse genomic locations
Source: BMC Microbiol. 2019 Mar 4;19:53. doi: 10.1186/s12866-019-1427-5 (PMC6399922; doi:10.1186/s12866-019-1427-5)
Supplement: Supplementary file 5 — Figure S3. Nucleotide alignment of the regions flanking regA in P. sordellii Tet P+ isolates. Sequences for the regA+ isolates SSCC32125, R32462, JGS6961 and SSCC18392 are compared to the regA− isolate W2945. The direct repeats flanking regA are shown in bold red font and indicated by a black arrow beneath the sequence. The coding region of regA is colored purple. A gap in the alignment representing the majority of regA is indicated by three large dots. The start and stop codons of regA are in bold and annotated on the sequence. The regA− isolates W3026, AM370 and W2922 have not been included in this alignment; however, a single site identical to the repeat is present for this isolate, equidistant to Tet P, as for W2945. (PDF 238 kb) [file 12866_2019_1427_MOESM5_ESM.pdf]

```
W2945           CTAAGATGACTTTCCCTCATCAACTTATGAAGTTAATTCTTTTAGAACAGGTTTATAGAT  60
JGS6961         CTAAGATGACTTTCCCTCATCAACTTATGAAGTTAATTCTTTTAGAACAGGTTTATAGAT  60
SSCC18392       CTAAGATGACTTTCCCTCATCAACTTATGAAGTTAATTCTTTTAGAACAGGTTTATAGAT  60
SSCC32135       CTAAGATGACTTTCCCTCATCAACTTATGAAGTTAATTCTTTTAGAACAGGTTTATAGAT  60
R32462          CTAAGATGACTTTCCCTCATCAACTTATGAAGTTAATTCTTTTAGAACAGGTTTATAGAT  60
                ************************************************************

W2945           CTTTTAGAATAAATAACAATGAACCGTATCACAAGTAAG---------------------  159
JGS6961         CTTTTAGAATAAATAATAATGAACCGTATCACAAGTAAGGTAGCGCGGATGATAAGAGTA  180
SSCC18392       CTTTTAGAATAAATAACAATGAACCGTATCACAAGTAAGGTAGCGCGGATGATAAGAGTA  180
SSCC32135       CTTTTAGAATAAATAACAATGAACCGTATCACAAGTAAGGTAGCGCGGATGATAAGAGTA  180
R32462          CTTTTAGAATAAATAACAATGAACCGTATCACAAGTAAGGTAGCGCGGATGATAAGAGTA  180
                **************** **********************
                                    ------------------->

W2945           ------------------------------------------------------------  159
JGS6961         AAAATTTAGATATATAATTTAGATCTTAGTTATACTTAAAAAATAATTAGTACGATAATA  240
SSCC18392       AAAATTTAGATATATAATTTAGATCTTAGTTATACTTAAAAAATAATTAGTACGATAATA  240
SSCC32135       AAAATTTAGATATATAATTTAGATCTTAGTTATACTTAAAAAATAATTAGTACGATAATA  240
R32462          AAAATTTAGATATATAATTTAGATCTTAGTTATACTTAAAAAATAATTAGTACGATAATA  240

W2945           ------------------------------------------------------------  159
JGS6961         TATGTATAGATAATATCGATAAAAGTATATTACTAAAAATTATAATTGTTAGTATGAGGA  300
SSCC18392       TATGTATAGATAATATCGATAAAAGTATATTACTAAAAATTATAATTGTTAGTATGAGGA  300
SSCC32135       TATGTATAGATAATATCGATAAAAGTATATTACTAAAAATTATAATTGTTAGTATGAGGA  300
R32462          TATGTATAGATAATATCGATAAAAGTATATTACTAAAAATTATAATTGTTAGTATGAGGA  300

W2945           ------------------------------------------------------------  159
JGS6961         GTGATTATACATGGATATGCTTACCAATTTCAATAAAGTTATGGAGTATATAGAAATGCA  420
SSCC18392       GTGATTATACATGGATATGCTTACCAATTTCAATAAAGTTATGGAGTATATAGAAATGCA  420
SSCC32135       GTGATTATACATGGATATGCTTACCAATTTCAATAAAGTTATGGAGTATATAGAAATGCA  420
R32462          GTGATTATACATGGATATGCTTACCAATTTCAATAAAGTTATGGAGTATATAGAAATGCA  420
                          START

                                   • • •

W2945           ------------------------------------------------------------  159
JGS6961         TGTTTATAGCGAAATTTGGATTGGGGTAAAAGAAAAGTAATTAGAACTGAAATTTTATTC  1200
SSCC18392       TGTTTATAGCGAAATTTGGATTGGGGTAAAAGAAAAGTAATTAGAACTGAAATTTTATTC  1200
SSCC32135       TGTTTATAGCGAAATTTGGATTGGGGTAAAAGAAAAGTAATTAGAACTGAAATTTTATTC  1200
R32462          TGTTTATAGCGAAATTTGGATTGGGGTAAAAGAAAAGTAATTAGAACTGAAATTTTATTC  1200
                                                    STOP

W2945           ------------------------------------------------------------  159
JGS6961         TATATCACAGTTAAAGGTACCTTGAATAATTTAAGGTGCCTTTTGATACTTATTTAGAAT  1260
SSCC18392       TATATCACAGTTAAAGGTACCTTGAATAATTTAAGGTGCCTTTTGATACTTATTTAGAAT  1260
SSCC32135       TATATCACAGTTAAAGGTACCTTGAATAATTTAAGGTGCCTTTTGATACTTATTTAGAAT  1260
R32462          TATATCACAGTTAAAGGTACCTTGAATAATTTAAGGTGCCTTTTGATACTTATTTAGAAT  1260

W2945           -----------------------------TGCGAACTTTACCAAAATTGGATATATAAAT  190
JGS6961         AACTATTAAGGAACCGTATCACAAGTAAGTGCGAACTTTACCAAAATTGGATATATAAAT  1320
SSCC18392       AACTATTAAGGAACCGTATCACAAGTAAGTGCGAACTTTACCAAAATTGGATATATAAAT  1320
SSCC32135       AACTATTAAGGAACCGTATCACAAGTAAGTGCGAACTTTACCAAAATTGGATATATAAAT  1320
R32462          AACTATTAAGGAACCGTATCACAAGTAAGTGCGAACTTTACCAAAATTGGATATATAAAT  1320
                          ------------------->*******************************

W2945           GAAAGGTAGGTCTGCTTTAATTCTGCAATGAAACTCACAAAAATCTAAAATTTGAATTTT  250
JGS6961         GAAAGGTAGGTCTGCTTTAATTCTGCAATGAAACTCACAAAAATCTAAAATTTGAATTTT  1380
SSCC18392       GAAAGGTAGGTCTGCTTTAATTCTGCAATGAAACTCACAAAAATCTAAAATTTGAATTTT  1380
SSCC32135       GAAAGGTAGGTCTGCTTTAATTCTGCAATGAAACTCACAAAAATCTAAAATTTGAATTTT  1380
R32462          GAAAGGTAGGTCTGCTTTAATTCTGCAATGAAACTCACAAAAATCTAAAATTTGAATTTT  1380
                ************************************************************
```
