# Supplementary material for: Paeniclostridium sordellii and Clostridioides difficile encode similar and clinically relevant tetracycline resistance loci in diverse genomic locations
Source: BMC Microbiol. 2019 Mar 4;19:53. doi: 10.1186/s12866-019-1427-5 (PMC6399922; doi:10.1186/s12866-019-1427-5)
Supplement: Supplementary file 8 — Table S3. Oligonucleotide primers used in PCR. (+) forward primer, (−) reverse primer. (PDF 89 kb) [file 12866_2019_1427_MOESM8_ESM.pdf]

Primer	Sequence (5’-3’)	Use
DLP134	CAGAGGATAGAAGCAGGGGATATAGG	Internal *P. sordellii tetB*(P) (+), generation of probe
DLP101	CTAATTGCGTTTAATTTATGAAGCAAATAC	Internal *P. sordellii tetB*(P) (-), generation of probe
DLP104	GGTGGTTGATAGAGTTGGAGGTATTG	Internal *P. sordellii tetA*(P) (+), screening of transconjugants
DLP105	TTGCTTCCTGCATCATATACTTCTTG	Internal *P. sordellii tetB*(P) (-), screening of transconjugants
JRP2094	CACAGATTGTATGGGGATTAGG	Internal *C. perfringens tetA*(P) (+), generation of probe
JRP2095	CATTTATAGAAAGCACAGTAGC	Internal *C. perfringens tetA*(P) (-), generation of probe
JRP4589	TTACAGTTCAAAACCCAACCTATGG	Internal *sdl* (+), generation of probe, screening of transconjugants
JRP4590	TGCAGCTTGTACATCTTTGCTCTTA	Internal *sdl* (-), generation of probe, screening of transconjugants
JRP2873	GTGAGGTTATGTTAATTATATGGTATAATTTCAATGC	Internal *C. perfringens plc* (+), screening of transconjugants
JRP2874	AGTTACAATCATAGCATGAGTTCCTGTTCC	Internal *C. perfringens plc* (-), screening of transconjugants
